# Fish Hydrolysate Supplementation Containing n-3 Long Chain Polyunsaturated Fatty Acids and Peptides Prevents LPS-Induced Neuroinflammation

**DOI:** 10.3390/nu13030824

**Published:** 2021-03-02

**Authors:** Mathilde Chataigner, Marie Martin, Céline Lucas, Veronique Pallet, Sophie Layé, Alexis Mehaignerie, Elodie Bouvret, Anne-Laure Dinel, Corinne Joffre

**Affiliations:** 1 Université de Bordeaux, INRAE, Bordeaux INP, NutriNeuro, 146 rue Léo Saignat, 33076 Bordeaux, France; mathilde@abyss-ingredients.com (M.C.); marie.martin33124@gmail.com (M.M.); Veronique.Pallet@enscbp.fr (V.P.); sophie.laye@inrae.fr (S.L.); anne-laure.dinel@inrae.fr (A.-L.D.); 2 Abyss Ingredients, 56850 Caudan, France; alexis@abyss-ingredients.com (A.M.); elodie@abyss-ingredients.com (E.B.); 3 NutriBrain Research and Technology Transfer, NutriNeuro, 146 rue Léo Saignat, 33076 Bordeaux, France; celine.lucas@nutribrain.fr

**Keywords:** low molecular weight peptides, docosahexaenoic acid (DHA), microglia, oxylipins, resolution of inflammation, fish byproducts

## Abstract

Neuroinflammation constitutes a normal part of the brain immune response orchestrated by microglial cells. However, a sustained and uncontrolled production of proinflammatory factors together with microglial activation contribute to the onset of a chronic low-grade inflammation, leading to neuronal damage and cognitive as well as behavioral impairments. Hence, limiting brain inflammatory response and improving the resolution of inflammation could be particularly of interest to prevent these alterations. Dietary n-3 long chain polyunsaturated fatty acids (LC-PUFAs) and low molecular weight peptides are good candidates because of their immunomodulatory and proresolutive properties. These compounds are present in a fish hydrolysate derived from marine-derived byproducts. In this study, we compared the effect of an 18-day supplementation with this fish hydrolysate to a supplementation with docosahexaenoic acid (DHA) on lipopolysaccharide (LPS)-induced inflammation in mice. In response to peripherally injected LPS, the fish hydrolysate supplementation decreased the hippocampal mRNA expression of the proinflammatory cytokines IL-6 (*p* < 0.001), IL-1β (*p* = 0.0008) and TNF-α (*p* < 0.0001), whereas the DHA supplementation reduced only the expression of IL-6 (*p* = 0.004). This decline in proinflammatory cytokine expressions was associated with an increase in the protein expression of IκB (*p* = 0.014 and *p* = 0.0054 as compared to the DHA supplementation and control groups, respectively) and to a modulation of microglial activation markers in the hippocampus. The beneficial effects of the fish hydrolysate could be due in part to the switch of the hippocampal oxylipin profile towards a more anti-inflammatory profile as compared to the DHA supplementation. Thus, the valorization of fish byproducts seems very attractive to prevent and counteract neuroinflammation.

## 1. Introduction

Neuroinflammation constitutes a normal part of the brain immune response. It remains a crucial mechanism of defense against pathogens and is involved in response to injury as well as wound healing [1,2]. Microglial cells, the immune cells of the central nervous system (CNS), orchestrate the immune response. Once activated, they display an activated morphology, proliferate and produce pro- and anti-inflammatory cytokines, including interleukin (IL)-1β, IL-6, tumor necrosis factor (TNF)-α, as well as growth factors such as brain-derived neurotrophic factor (BDNF) to ensure the return to homeostasis [3,4,5,6,7,8]. However, a sustained and uncontrolled production of proinflammatory factors together with microglial activation contribute to the onset of chronic low-grade inflammation, leading to neuronal damage, as observed in neurodegenerative diseases and aging [9,10,11]. Indeed, it has been proposed that some diseases that are associated with chronic inflammation could be explained by dysregulated resolution of inflammation as much as by ongoing proinflammatory processes [12]. The functional consequences of dysregulated inflammation include cognitive impairment as well as behavioral alterations, notably depression [13] that could seriously impact the quality of life. Hence, limiting excessive brain inflammatory responses and improving the resolution of inflammation is of importance. 

Nutrition appears as an innovative strategy to prevent neuroinflammation-related impairments. Notably, n-3 long chain polyunsaturated fatty acids (LC-PUFAs) and low molecular weight peptides are of interest for their immunomodulatory and proresolutive properties. 

n-3 LC-PUFAs, including docosahexaenoic acid (DHA) and eicosapentaenoic acid (EPA), display powerful anti-inflammatory and proresolutive properties. Indeed, they regulate the release of proinflammatory mediators as evidenced in clinical and preclinical studies, and in vitro [14,15,16] and are precursors of oxylipins called specialized proresolving mediators (SPMs) [15,17,18,19,20,21,22]. They also affect the synthesis of oxylipins derived from arachidonic acid (AA) that are mainly proinflammatory [20,23,24]. The level of oxylipins are regulated by external factors such as proinflammatory stimuli and dietary supply. Indeed, central and peripheral expression of oxylipins’ synthesis enzymes (cyclooxygenases, lipoxygenases, cytochrome P450) as well as oxylipins levels are tightly regulated by inflammatory stimuli [15,20,25,26,27,28,29]. In addition, n-3 LC-PUFA supplementations increase cerebral and peripheral oxylipins derived from DHA and EPA, decrease lipid mediators derived from AA and regulate oxylipins’ synthesis enzymes [20,23,24,28,30,31,32]. 

Low molecular weight peptides (<1000 Da) contained in protein hydrolysates are also nutrients of interest for their central and peripheral anti-inflammatory properties [33,34]. Indeed, bioactive peptides derived from various food sources inhibit the proinflammatory c-Jun N-terminal kinase/mitogen-activated protein kinase (JNK/MAPK) and nuclear factor kappa B (NF-κB) pathways in vivo and in vitro [35,36,37,38]. In mice, peptides from milk reduced the expression of inflammatory factors such as TNF-α, monocyte chemoattractant protein-1 (MCP-1/CCL2) or inducible nitric oxide synthase (iNOS) in the hippocampus in a model of Alzheimer’s disease [39]. In D-galactose-induced mice ageing, a hydrolysate obtained from lantern fish and containing active di- and tripeptides induced an increased level of BDNF [40]. In vitro, in human primary monocytes and murine macrophages, salmon and lupine-derived peptides inhibited the production of nitric oxide (NO), prostaglandin (PG) E2 and proinflammatory cytokines including TNF-α, IL-6 and IL-1β [41,42]. Moreover, peptides from soy and milk reduced the peripheral expression of proinflammatory factors such as TNF-α, IL-6, IL-1β, interferon-γ and IL-17 in mice colon and abdominal aorta [37,43]. 

Combining low molecular weight peptides and n-3 LC-PUFAs could be relevant to prevent neuroinflammation since we already demonstrated a beneficial effect of this association in age-related cognitive decline [44]. However, to our knowledge, no studies demonstrated its anti-inflammatory properties. Thus, we explored in mice the effect of a dietary supplementation with a fish hydrolysate, obtained by means of a sustainable developmental approach from marine-derived byproducts, on LPS-induced inflammation and compared it to a DHA supplementation whose anti-inflammatory properties have already been described in the literature. 

## 2. Materials and Methods

### 2.1. Animals and Treatments

All experiments were performed with 7-week-old C57Bl/6J mice obtained from Janvier Labs (Le Genest-Saint-Isle, France). Mice were maintained under standard housing conditions, on cellulose litter, in a temperature (21 ± 2 °C) and humidity (40%) controlled environment with a 12 h light/dark cycle (9:00–21:00) and with ad libitum access to water and A04 food (Safe, Augy, France). A total of 33 animals were used in this study. This study was approved by the national ethical committee for the care and use of animals (approval ID A16320) and conducted in accordance with the EU Directive 2010/63/EU for animal experiments. Mice received by gavage via a gastric tube (Ecimed, Boissy-Saint-Léger, France) during 18 days 150 µL of supplement containing the fish hydrolysate (from the Brain Booster consortium, n = 10) or 10mg of DHA alone (Polaris, Quimper, France, n = 12) or peanut oil and water for control (n = 11). DHA and fish hydrolysate were diluted in peanut oil or water respectively. The fish hydrolysate was obtained from marine byproducts. The 150 µL fish hydrolysate supplement contained 5 mg of low molecular weight peptides (<1000 Da) and 143 µg of DHA (Table 1). This dose was determined by a literature review [45,46]. At the end of the supplementation period, a solution of LPS (lipopolysaccharide; Escherichia coli, 0127: B8, Sigma-Aldrich, Lyon, France) at a dose of 125 µg/kg (diluted in saline solution NaCl 0.9%) was intraperitoneally injected in 4 fish hydrolysate mice, 6 DHA mice and 6 control mice [20,47]. Six fish hydrolysate mice, 6 DHA mice and 5 control mice received an injection of saline solution. 

Two hours after the injection, mice were anesthetized by isoflurane inhalation and euthanized. After transcardiac perfusion of phosphate buffered saline (PBS), brain structures of interest (hippocampus and whole brain cortex) were isolated and stored at −80 °C until analysis. 

### 2.2. Quantitative Real Time PCR

mRNA expression was analyzed on hippocampus as described by Rey et al. (2019) [20]. Total RNAs were extracted using the extraction protocol of TRIzol (Invitrogen, Life Technologies, Villebon sur Yvette, France). The purity and the amount of RNA of each sample were determined using Nanodrop spectrophotometer (Nanodrop One, Life Technologies, France). Two micrograms of RNA were reverse transcribed to synthesize cDNA (complementary DNA) using Superscript III (Invitrogen, Life Technologies, France). As previously described, cDNAs were amplified by PCR using Taqman® primers specific to target genes studied [20,47]. We focused on IL-6 (Mm00446190_m1), IL-1β (Mm00434228_m1), TNF-α (Mm00443258_m1), cluster of differentiation 206 (CD206; Mm00485148_m1), Arginase 1 (Arg1; Mm00475988_m1), suppressor of cytokine signaling 3 (SOCS3; Mm00545913_s1), transforming growth factor β1 (TGF-β1; Mm01178820_m1), cluster of differentiation 68 (CD68; Mm03047343_m1), cluster of differentiation 86 (CD86; Mm00444540_m1), cluster of differentiation 36 (CD36; Mm00432403_m1), cluster of differentiation 11b (CD11b; Mm00434455_m1), toll-like receptor 4 (TLR4; Mm00434455_m1), C-C Motif Chemokine Ligand 2 (CCL2; Mm00441242_m1), BDNF (Mm04230607_s1), tyrosine receptor kinase B (TrkB; Mm00435422_m1), nerve growth factor (NGF; Mm00443039_m1), tyrosine receptor kinase A (TrkA; Mm01219406_m1), cyclooxygenase-2 (COX-2; Mm00478374_m1), 15-lipoxygenase (15-LOX; Mm00507789_m1), 5-lipoxygenase (5-LOX; Mm01182747_m1) and beta-2 microglobulin (B2m; Mm00437762_m1) as reference gene. These genes are involved as modulators of inflammatory response [7,16]. Fluorescence was measured using an ABI PRISM 7500-sequence detection system (Applied Biosystems, Villebon sur Yvette, France) and final quantification was analyzed by the comparative threshold cycle (Ct) method. Results are expressed as relative fold change [20,47] to control target mRNA expression.

### 2.3. Western Blot

Protein expression was evaluated on hippocampus. Proteins were extracted using the extraction protocol of Simões et al. (2013) [48]. Protein concentration was determined by bicinchoninic acid protein assay (Interchim, Montlucon, France) according to the protocol. For analysis, proteins were resolved on 12% sodium dodecyl sulfate-polyacrylamide gel and transferred to nitrocellulose membranes. Membranes were incubated with different primary antibodies: anti-BDNF (ab108319, rabbit, Abcam, Cambridge, UK), anti-TrkB (4603, rabbit, Cell Signaling, Leiden, The Netherlands), anti-NF-κB (14220-1-AP, rabbit, ProteinTECH, Manchester, UK), anti-IκB (51066-1-AP, rabbit, ProteinTECH, Manchester, UK) and anti-GAPDH as housekeeping protein (51745, rabbit, Cell Signaling, Leiden, The Netherlands). These primary antibodies were detected with appropriated donkey horseradish peroxidase-conjugated secondary antibodies (711-035-152, Jackson Immunoresearch, Westgrove, PA, USA). The membranes were incubated with a peroxidase revealing solution (SuperSignal West Dura, ThermoFisher, Waltham, MA, USA) and were revealed using ChemiDoc MP (Biorad, Hercules, CA, USA). Proteins of interest were normalized to GAPDH and results are expressed as relative expression.

### 2.4. Lipid Analysis

Lipids from the cortex were extracted and fatty acids were transmethylated as previously described [49,50,51]. Fatty acid methyl esters were analyzed using gas chromatography on a Hewlett Packard Model 5890 series II gas chromatograph (Palo Alto, CA, USA) equipped with a split/spitless injector, a flame ionization detector (Palo Alto, CA, USA), and a CPSIL-88 column (100 m × 0.25 mm internal diameter; film thickness, 0.20 µm; Varian, Les Ulis, France). The carrier gas was hydrogen (inlet pressure, 210 kPa). The oven temperature was held at 60 °C for 5 min, increased to 165 °C at 15 °C/min and held for 1 min, and then to 225 °C at 2 °C/min and finally held at 225 °C for 17 min. The injector and the detector were maintained at 250 °C and 280 °C, respectively. Fatty acid methyl esters were identified by comparison with commercial standards. Fatty acid composition is expressed as a percentage of total fatty acids.

### 2.5. Oxylipin Quantification

The different metabolites derived from LA, AA, DHA and EPA were extracted from hippocampus and analyzed by mass spectrometry (LC-MS/MS) at METATOUL platform (MetaboHUB, INSERM UMR 1048, I2MC, Toulouse, France) as previously described by Le Faouder et al. (2013) [52]. Results are expressed as pg/mg of protein.

### 2.6. Statistical Analysis

Statistical analyses were performed using GraphPad Prism 7 (GraphPadSotfware, San Diego, CA, USA). The 3 groups were compared using a 2-way ANOVA test (with LPS and supplementation as factors) followed by an LSD Fisher *post hoc* test comparison when appropriate. 

Principal component analysis (PCA) was performed, using RStudio software (RStudio Desktop 1.3.1056, Boston, MA, USA). The number of components was selected using Cattell criterion. All the data were expressed as means ± SEM. Statistical significance was set at *p* < 0.05.

## 3. Results

### 3.1. Fish Hydrolysate Supplementation Decreases Proinflammatory Marker Expression and Prevents IκB Degradation in Response to LPS

**Figure 1:**
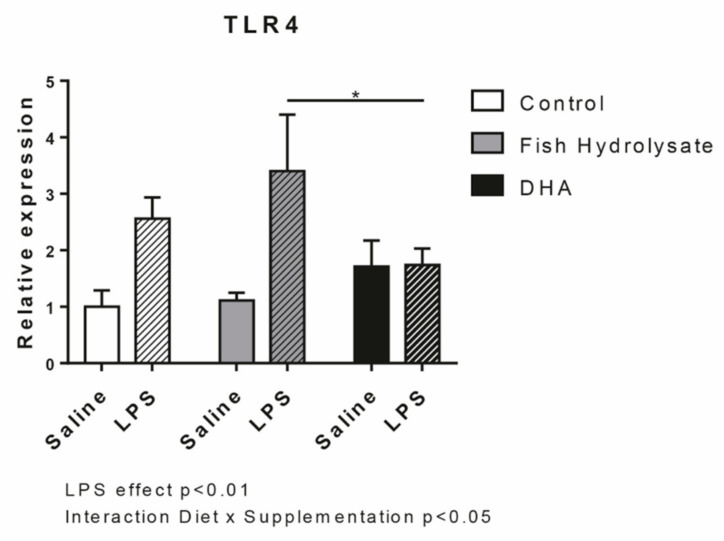
Expression of the lipopolysaccharide (LPS) receptor TLR4 in the hippocampus of mice fed orally with control solution (in white), fish hydrolysate (in grey) or DHA (in black) by daily gavage for 18 days, 2 h following LPS injection (125 µg/kg) (hatched bars) (* *p* < 0.05 by 2-way ANOVA and Fisher’s LSD *post hoc* test; n = 4–6 per group). Data are presented as mean ± SEM. DHA: docosahexaenoic acid; LPS: lipopolysaccharide.

We first evaluated the expression of LPS receptor, TLR4. As expected, LPS increased TLR4 expression (F_(1,27)_ = 13.36, *p* = 0.011) but no significant effect of both supplementations were observed. However, the interaction between LPS and supplementation was significant (F_(2,27)_ = 3.61, *p* = 0.0407). DHA supplementation reduced the LPS-induced expression of TLR4 compared to the fish hydrolysate supplementation (*p* = 0.0163) (Figure 1). 

**Figure 2:**
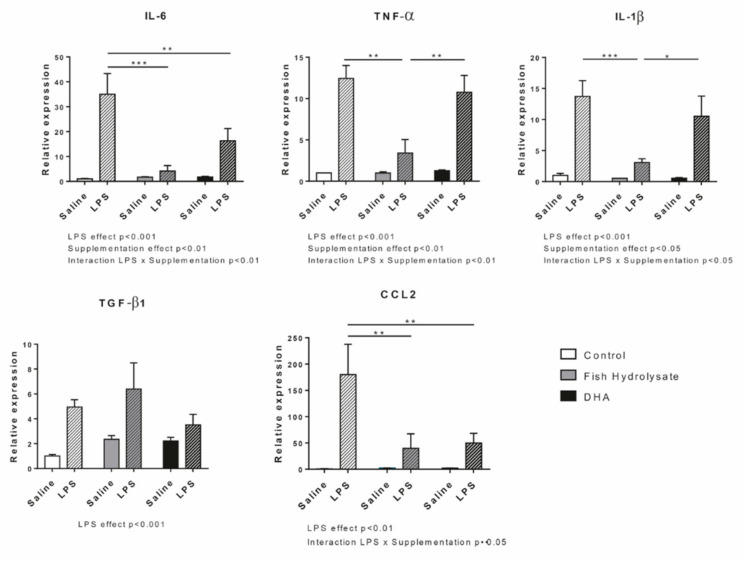
Expression of pro- and anti-inflammatory cytokines (IL-6, TNF-α, IL-1β, TGF-β1) and chemokine (CCL2) in the hippocampus of mice fed orally with control solution (in white), fish hydrolysate (in grey) or DHA (in black) by daily gavage for 18 days, 2 h following LPS injection (125 µg/kg) (hatched bars) (* *p* < 0.05, ** *p* < 0.01, *** *p* < 0.001 by 2-way ANOVA and Fisher’s LSD *post-hoc* test; n = 4–6 per group). Data are presented as mean ± SEM. DHA: docosahexaenoic acid; LPS: lipopolysaccharide.

We then determined the impact of both supplementations on pro- and anti-inflammatory cytokine and chemokine expression in response to LPS (Figure 2). As expected, LPS significantly increased the mRNA expression of IL-6 (F_(1,27)_ = 21.86, *p* < 0.0001), TNF-α (F_(1,27)_ = 56.96, *p* < 0.0001), IL-1β (F_(1,27)_ = 29.87, *p* < 0.0001), TGF-β1 (F_(1,25)_ = 19.87, *p* = 0.0002) and CCL2 (F_(1,25)_ = 12.16, *p* = 0.0018). Supplementations significantly modulated proinflammatory cytokine expression (IL-6: F_(2,27)_ = 5.595, *p* = 0.0093; TNF-α: F_(2,27)_ = 6.949, *p* = 0.0037 and IL-1β: F_(2,27)_ = 4.239, *p* = 0.0251) and tended to decrease CCL2 expression (F_(2,25)_ = 3.06, *p* = 0.065) but not the anti-inflammatory marker expression. Interestingly, the two-way ANOVA analysis revealed a significant interaction between LPS and supplementation factors for IL-6 (F_(2,27)_ = 6.195, *p* = 0.0061), TNF-α (F_(2,27)_ = 6.636, *p* = 0.0045), IL-1β (F_(2,27)_ = 3.673, *p* = 0.0388) and CCL2 (F_(2,25)_ = 3.18, *p* = 0.05) but not for TGF-β1. Indeed, IL-6 expression was significantly decreased by the fish hydrolysate and DHA supplementations (*p* < 0.001 and *p* = 0.0040, respectively) whereas TNF-α and IL-1β expression was only decreased by the fish hydrolysate supplementation (*p* < 0.0001 and *p* = 0.0008, respectively) in LPS-treated animals. Furthermore, LPS-induced IL-6 expression tended to be more decreased in animals supplemented with the fish hydrolysate as compared to animals treated by DHA (*p* = 0.0788). CCL2 expression was decreased by both fish hydrolysate (*p* = 0.0043) and DHA (*p* = 0.0032) supplementations in LPS-treated mice. 

**Figure 3:**
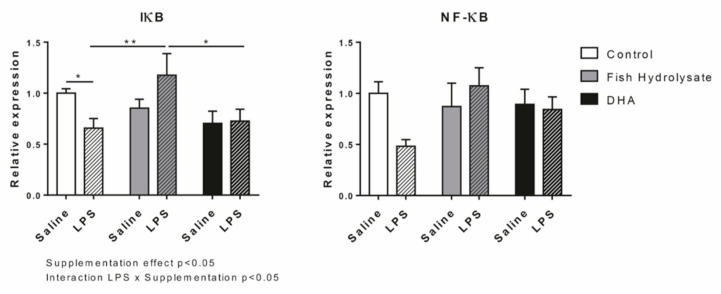
Protein expression of proinflammatory factors IκB and NF-κB in the hippocampus of mice fed orally with control solution (in white), fish hydrolysate (in grey) or DHA (in black) by daily gavage for 18 days, 2 h following LPS injection (125 µg/kg) (hatched bars) (* *p* < 0.05, ** *p* < 0.01 by 2-way ANOVA and Fisher’s LSD *post hoc* test; n = 4–6 per group). Data are presented as mean ± SEM. DHA: docosahexaenoic acid; LPS: lipopolysaccharide.

Finally, we examined protein expressions of IκB and NF-κB involved in the regulation of the expression of these inflammatory factors (Figure 3). No significant effect of LPS was observed on both factors. The supplementations affected the protein expression of IκB (F_(2,27)_ = 3.41, *p* = 0.0479) and an interaction between supplementation and LPS was revealed only for IκB (F_(2,27)_ = 4.03, *p* = 0.0293). Thus, in inflammatory condition, the protein expression of IκB was significantly increased by the fish hydrolysate supplementation as compared to the DHA supplementation (*p* = 0.0141) and to the control group (*p* = 0.0054).

### 3.2. Fish Hydrolysate Supplementation Modulates Microglial Activation Markers in Response to LPS 

**Figure 4:**
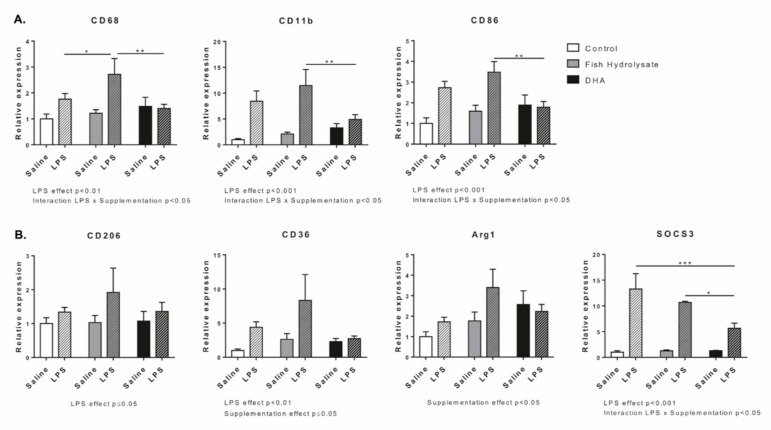
Expression of M1 (**A**) (CD68, CD11b and CD86) and M2 (**B**) (CD206, CD36, Arg1 and SOCS3) microglial markers in the hippocampus of mice fed orally with control solution (in white), fish hydrolysate (in grey) or DHA (in black) by daily gavage for 18 days, 2 h following LPS injection (125 µg/kg) (hatched bars) (* *p* < 0.05, ** *p* < 0.01, *** *p* < 0.001 by 2-way ANOVA and Fisher’s LSD *post hoc* test; n = 4–6 per group). Data are presented as mean ± SEM. DHA: docosahexaenoic acid; LPS: lipopolysaccharide.

We evaluated the expression of different markers of microglial activation (Figure 4A). LPS significantly increased the mRNA expression of CD68 (F_(1,27)_ = 9.86, *p* = 0.0041), CD11b (F_(1,25)_ = 25.58, *p* < 0.0001) and CD86 (F_(1,27)_ = 15.28, *p* = 0.0006). Supplementation had no effect on CD68, CD11b and CD86. However, the two-way ANOVA analysis revealed a significant interaction between LPS and supplementation for CD68 (F_(2,27)_ = 2.41, *p* = 0.0326), CD11b (F_(2,25)_ = 3.84, *p* = 0.0351) and CD86 (F_(2,27)_ = 4.83, *p* = 0.0161). Compared to DHA supplementation, the fish hydrolysate supplementation significantly increased CD68 (*p* = 0.0045), CD11b (*p* = 0.0052) and CD86 (*p* = 0.0045) expression in response to LPS. 

Then, we determined the expression of anti-inflammatory markers (Figure 4B). LPS significantly increased the mRNA expression of CD206 (F_(1,27)_ = 4.19, *p* = 0.05), CD36 (F_(1,26)_ = 9.23, *p* = 0.0054) and SOCS3 (F_(1,24)_ = 42.15, *p* < 0.0001). Supplementation affected the mRNA expression of Arg1 (F_(2,26)_ = 3.79, *p* < 0.0358) and CD36 (F_(2,26)_ = 3.22, *p* = 0.05) and tended to modulate SOCS3 (F_(2,24)_ = 3.003, *p* = 0.069). An interaction between LPS and supplementation was highlighted for SOCS3 (F_(2,24)_ = 3.46, *p* = 0.0480). Indeed, DHA supplementation decreased SOCS3 expression in LPS-treated animals compared to control (*p* = 0.0011) and fish hydrolysate supplemented animals (*p* = 0.05).

### 3.3. Fish Hydrolysate Supplementation Prevents LPS-Induced Changes in the Expression of Neurotrophins 

**Figure 5:**
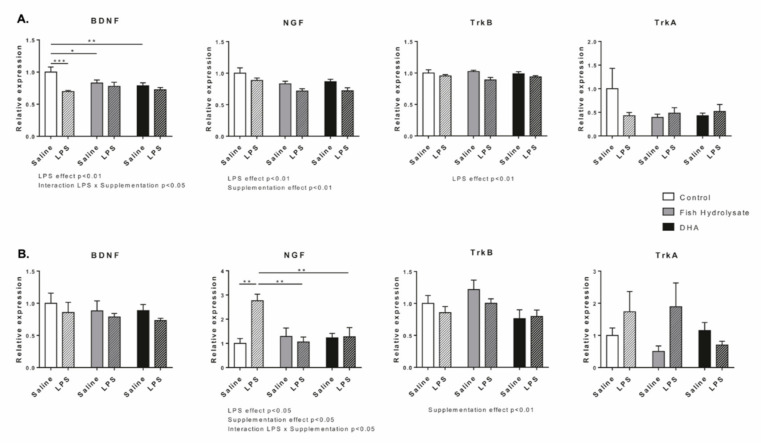
Gene (**A**) and protein (**B**) expression of neurotrophins (BDNF, NGF) and their receptors (TrkB and TrkA) in the hippocampus of mice fed orally with control solution (in white), fish hydrolysate (in grey) or DHA (in black) by daily gavage for 18 days, 2 h following LPS injection (125 µg/kg) (hatched bars) (* *p* < 0.05, ** *p* < 0.01, *** *p* < 0.001 by 2-way ANOVA and Fisher’s LSD *post-hoc* test; n = 4–6 per group). Data are presented as mean ± SEM. DHA: docosahexaenoic acid; LPS: lipopolysaccharide.

We investigated the effect of supplementations on the mRNA expression of neurotrophins BDNF, NGF and their respective receptors TrkB and TrkA (Figure 5a). LPS significantly decreased the mRNA expression of BDNF (F_(1,26)_ = 11.13, *p* = 0.0026), NGF (F_(1,27)_ = 9.12, *p* = 0.0055) and TrkB (F_(1,27)_ = 8.80, *p* = 0.0062) but had no effect on TrkA expression. Supplementations decreased mRNA expression of NGF (F_(2,27)_ = 6.61, *p* = 0.0046). Interestingly, the basal mRNA expression of BDNF was decreased in fish hydrolysate (F_(2,26)_ = 3.81, *p* = 0.0354, *p* = 0.0222) and DHA supplemented animals ((F_(2,26)_ = 3.81, *p* = 0.0354), *p* = 0.0054). Moreover, in response to LPS, BDNF mRNA expression was blunted in animals fed with the fish hydrolysate or DHA (F_(2,26)_ = 3.81, *p* = 0.0354).

Then, we measured the protein expression of these factors (Figure 5b). LPS significantly impacted the expression of NGF (F_(1,27)_ = 4.79, *p* = 0.0374) but had no effect on BDNF, TrkB and TrkA expression. Supplementations significantly affected protein expression of NGF (F_(2,27)_ = 3.49, *p* = 0.0447) and TrkB (F_(2,27)_ = 3.61, *p* = 0.0407) but not BDNF and TrkA. The two-way ANOVA showed that the protein expression of NGF significantly increased in the control group in response to LPS whereas it remained stable (compared to saline) in fish hydrolysate and DHA groups (F_(2,27)_ = 6.73, *p* = 0.0043; *p* = 0.0026). Moreover, in LPS-treated animals, fish hydrolysate and DHA supplementations significantly decreased NGF expression as compared to the control group (F_(2,27)_ = 6.73, *p* = 0.0043, *p* = 0.0073 and *p* = 0.0089, respectively).

### 3.4. Fish Hydrolysate and DHA Supplementations Affect Cortical Fatty Acid Composition 

We measured the impact of both supplementations on fatty acid composition in the cortex (Table 2). Supplementations significantly modulated total n-6 and total n-3 PUFA levels (total n-6 PUFAs: F_(2,27)_ = 17.22, *p* < 0.0001 and total n-3 PUFAs: F_(2,27)_ = 5.48, *p* = 0.01). Almost all n-6 and n-3 long chain PUFAs were impacted: 20:3 n-6 (F_(2,26)_ = 23.39, *p* < 0.0001), 20:4 n-6 (F_(2,27)_ = 23.70, *p* < 0.0001), 22:4 n-6 (F_(2,26)_ = 6.05, *p* = 0.0069), 22:5 n-6 (F_(2,25)_ = 13.13, *p* = 0.0001), 20:5 n-3 (F_(2,27)_ = 19.59, *p* < 0.0001), 22:5 n-3 (F_(2,27)_ = 64.00, *p* < 0.0001) and 22:6 n-3 (F_(2,27)_ = 4.72, *p* = 0.0175). These changes modulated the n-6/n-3 ratio (F_(2,27)_ = 17.72, *p* < 0.0001). LPS treatment didn’t have any effect on total n-6 and n-3 PUFA content. However, LPS affected 4 fatty acids, among them three minor PUFAs: 22:4 n-6 (F_(1,26)_ = 5.96, *p* = 0.0218), 20:2 n-6 (F_(1,27)_ = 4.50, *p* = 0.0432) and 20:3 n-9 (F_(1,27)_ = 8.68, *p* = 0.0066) and one major saturated fatty acid: 18:0 (F_(1,27)_ = 19.93, *p* = 0.0001).

### 3.5. Fish Hydrolysate Supplementation Affects Hippocampal Oxylipin Concentration in LPS-Treated Animals

We evaluated the impact of the supplementations on the concentration of oxylipins derived from n-6 or n-3 PUFAs in the hippocampus in basal condition or in response to LPS (Table 3). Many oxylipins derived from AA were detected: thromboxanes and prostaglandins (TxB2, 6kPGF1α, PGF2-α, PGE2, PGD2, PGA1, 15dPGJ2, 8isoPGA2), lipoxins (LxA4 and LxB4), epoxy fatty acids (14,15-EET, 8,9-EET, 5,6-EET) and hydroxy fatty acids (15-HETE, 8-HETE, 12-HETE, 5-HETE, 5-oxoETE). We also detected hydroxy fatty acids derived from linoleic acid (LA) (13-HODE and 9-HODE) and hydroxy fatty acids derived from DHA (17-HDoHE, 14-HDoHE and 7-MaR1).

The two-way ANOVA analysis revealed that most of the significant effects were due to LPS treatment. Indeed, LPS impacted the amounts of oxylipins derived from AA: PGE2 (F_(1,27)_ = 16.76, *p* = 0.0003), PGD2 (F_(1,27)_ = 11.75, *p* = 0.002), PGA1 (F_(1,26)_ = 31.51, *p* < 0.0001), 15dPGJ2 (F_(1,27)_ = 6.83, *p* = 0.0145), 8isoPGA2 (F_(1,27)_ = 6.29, *p* < 0.0184), LxA4 (F_(1,27)_ = 21.68, *p* < 0.0001), 15-HETE (F_(1,27)_ = 27.48, *p* < 0.0001), 8-HETE (F_(1,27)_ = 19.65, *p* = 0.0001), 12-HETE (F_(1,27)_ = 22.45, *p* < 0.0001), 5-HETE (F_(1,26)_ = 5.56, *p* = 0.0262), 5-oxoETE (F_(1,26)_ = 4.99, *p* = 0.0342). The same results were found for those derived from LA: 13-HODE (F_(1,26)_ = 11.09, *p* = 0.0026) and 9-HODE (F_(1,26)_ = 15.84, *p* = 0.0005) and also for those derived from DHA: 17-HDoHE (F_(1,27)_ = 19.85, *p* = 0.0001), 14-HDoHE (F_(1,27)_ = 16.10, *p* = 0.0004) and 7-MaR1 (F_(1,27)_ = 11.91, *p* = 0.0019). The supplementations modulated the amounts of oxylipins derived from AA: PGA1 (F_(2,26)_ = 8.60, *p* = 0.0014), LxB4 (F_(2,27)_ = 3.40, *p* = 0.0483), 5-HETE (F_(2,26)_ = 3.95, *p* = 0.0318), 5-oxoETE (F_(2,26)_ = 6.00, *p* = 0.0072) and of 7-MaR1 (F_(2,27)_ = 4.05, *p* = 0.0289) derived from DHA.

However, an interaction LPS x supplementation was observed for these oxylipins: those derived from AA: PGE2 (F_(2,27)_ = 4.46, *p* = 0.0211), PGD2 (F_(2,27)_ = 3.95, *p* = 0.0314), PGA1 (F_(2,26)_ = 8.60, *p* = 0.0014), LxA4 (F_(2,27)_ = 6.77, *p* = 0.0041), 15-HETE (F_(2,27)_ = 8.44, *p* = 0.0014), 8-HETE (F_(2,27)_ = 7.13, *p* = 0.0033), 12-HETE (F_(2,27)_ = 6.66, *p* = 0.0044) and 5-oxoETE (F_(2,26)_ = 7.86, *p* = 0.0022); those derived from LA: 13-HODE (F_(2,26)_ = 5.12, *p* = 0.0134) and 9-HODE (F_(2,26)_ = 6.12, *p* = 0.0066); and those derived from DHA: 17-HDoHE (F_(2,27)_ = 5.87, *p* = 0.0076), 14-HDoHE (F_(2,27)_ = 6.32, *p* = 0.0056) and 7-MaR1 (F_(2,27)_ = 4.05, *p* = 0.0289). Indeed, in saline conditions, the fish hydrolysate supplementation increased 17-HDoHE as compared to the DHA group (*p* = 0.0158). Moreover, DHA supplementation significantly increased 15-HETE, 8-HETE, 12-HETE, 5-oxoETE and 14-HDoHE levels as compared to the control (15-HETE: *p* = 0.0299, 14-HDoHE: *p* = 0.0363, 5-oxoETE: *p* = 0.0022) and fish hydrolysate supplementation (15-HETE: *p* = 0.0113, 12-HETE: *p* = 0.0376, 8-HETE: *p* = 0.0069, 14-HDoHE: *p* = 0.0031 and 5-oxoETE: *p* = 0.0007). In response to LPS, the fish hydrolysate supplementation increased PGE2 (*p* = 0.0023) and 12-HETE (*p* < 0.0001) as compared to saline-treated animals. It also increased PGA1, LxA4, 15-HETE, 8-HETE, 5-oxoETE, 13-HODE, 9-HODE, 14-HDoHE, 17-HDoHE and 7-MaR1 as compared to control (LxA4: *p* = 0.0366, 8-HETE: *p* = 0.0445, 14-HDoDE: *p* = 0.0252, 5-oxoETE: *p* = 0.0052, 13-HODE: *p* = 0.0069 and 9-HODE: *p* = 0.0084) and to DHA supplementation (LxA4: *p* = 0.0044, 8-HETE: *p* = 0.0210, 13-HODE: *p* = 0.0118, 17-HDoHE: *p* = 0.0332, 15-HETE: *p* = 0.0072, 9-HODE: *p* = 0.0062, PGA1: *p* < 0.0001 and 7-MaR1: *p* = 0.0007). Moreover, DHA supplementation decreased PGA1 (*p* = 0.0001), PGD2 (*p* = 0.0339) and 7-MaR1 (*p* = 0.05) levels as compared to control.

### 3.6. Multivariate Analysis Highlights the Separation of Oxylipin Profile in Function of the Supplementation in LPS-Treated Animals

**Figure 6:**
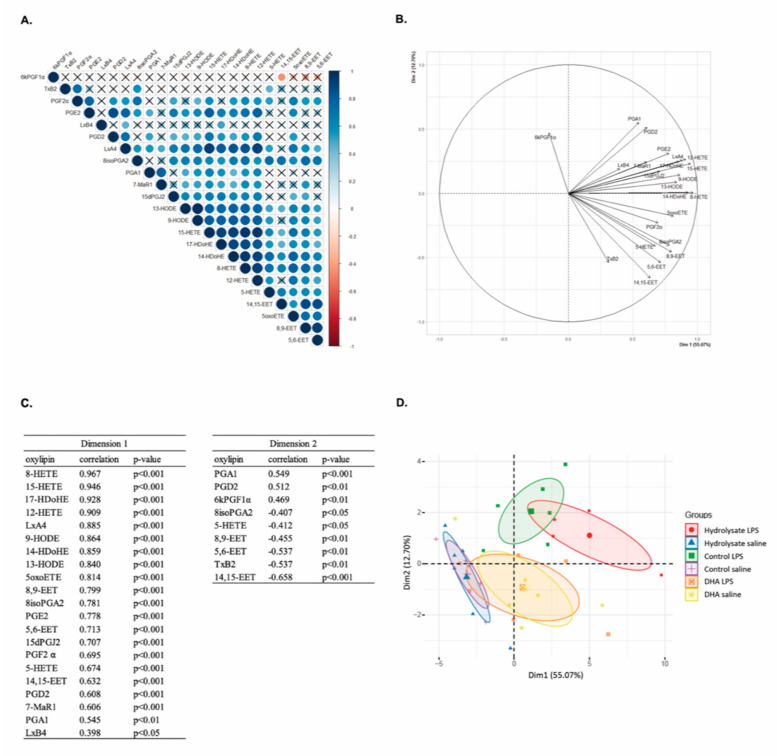
Multivariate analysis of oxylipins in the hippocampus of mice fed orally with control solution, fish hydrolysate or DHA by daily gavage for 18 days, 2 h following LPS injection (125 µg/kg)**.** (**A**) Correlation matrix of the 23 oxylipins (blue: positive correlation, red: negative correlation, X: no significant correlation). (**B**) Correlation circle from PCA. (**C**) Correlation between each variable and the principal component score from PCA (dimension 1 or dimension 2). (**D**) Individual map of PCA. 5-oxoETE: 5-oxo-eicosatetraenoic; 7-MaR1: 7(S)-maresin; EET: epoxy-eicosatrienoic acid; HDoHE: hydroxy-docosahexaenoic acid; HETE: hydroxy-eicosatetraenoic acid; HODE: hydroxy-octadecadienoic acid; Lx: lipoxin; PCA: principal component analysis; PG: prostaglandin; TxB2: thromboxane B2.

We performed multivariate analysis to evaluate if our groups could be differentiated by an oxylipin pattern. The 23 variables corresponding to the 23 oxylipins detected in the mice hippocampus were analyzed. The correlation matrix revealed the most correlated variables. Significant correlations were highlighted (*p* < 0.05) (Figure 6A): except 6kPGF1α, TxB2 and LxB4, other oxylipins were mostly positively correlated together. According to the correlation matrix, PCA analysis revealed that the oxylipins were mostly grouped (Figure 6B). Indeed, the first component “dim 1” explained 55.07% of the total variance and it showed a positive score for all the oxylipins except 6kPGF1α and TxB2. The component 2 “dim 2” explained 12.70% of the variance and revealed a positive score for PGA1, PGD2 and 6kPGF1α and a negative score for 8isoPGA2, 5-HETE, TxB2, 8,9-EET, 5,6-EET and 14,15-EET (Figure 6C).

PCA visualization of individuals revealed a separation between the different groups (Figure 6D). Indeed, in basal condition, the control and fish hydrolysate groups showed the same high and negative average score for component 1 whereas the DHA group revealed an average score close to 0. In response to LPS, the DHA group showed a similar profile to that in basal conditions whereas the control and fish hydrolysate groups revealed different profiles as compared to their basal condition but evolved in different ways. Indeed, the control group showed a higher positive average score for component 2 but a small positive average score for component 1 as compared to the fish hydrolysate group. Thus, according to the supplementations, LPS treatment caused a different oxylipin variation.

### 3.7. LPS-Induced Hippocampal Expression of Oxylipin Biosynthesis Enzymes Is Regulated by Supplementations

**Figure 7:**
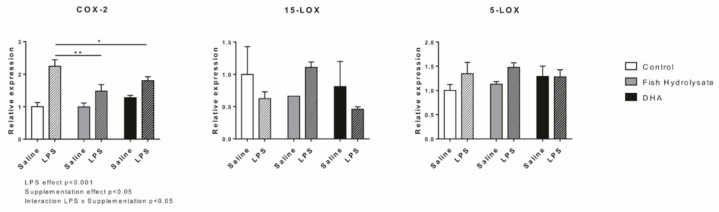
Expression of oxylipin biosynthesis enzymes (COX-2, 15-LOX and 5-LOX) in the hippocampus of mice fed orally with control solution, fish hydrolysate or DHA by daily gavage for 18 days, 2 h following LPS injection (125 µg/kg) (* *p* < 0.05, ** *p* < 0.01 by 2-way ANOVA and Fisher’s LSD *post hoc* test; n = 4–6 per group). Data are presented as mean ± SEM. DHA: docosahexaenoic acid; LPS: lipopolysaccharide.

Oxylipins are mainly generated through the actions of COX-2, 5-LOX and 15-LOX. Hippocampal expression of these enzymes was measured 2 h following LPS injection. LPS significantly increased mRNA expression of COX-2 (F_(1,27)_ = 40.6, *p* < 0.0001) but had no effect on 15-LOX and 5-LOX (15-LOX: F_(1,14)_ = 0.235, *p* = 0.635; 5-LOX: F_(1,25)_ = 2.289, *p* = 0.143). Both supplementations significantly decreased COX-2 expression (F_(2,27)_ = 3.753, *p* = 0.0375) and the interaction LPS x supplementations was significant (F_(2,27)_ = 4.432, *p* = 0.0217) (Figure 7). Indeed, the fish hydrolysate and DHA supplementations significantly reduced the LPS-induced expression of COX-2 (*p* = 0.0014 and *p* = 0.0294, respectively). No significant effects were found on 15-LOX and 5-LOX expressions. 

## 4. Discussion

This work provides evidence that dietary supplementation with fish hydrolysate reduces the LPS-induced proinflammatory cytokine expression and switch hippocampal oxylipin profile, thus counteracting neuroinflammation. 

Neuroinflammation is a normal part of the immune response against pathogen invasion or tissue injury [1,2]. The inflammatory response is an active process, limited in time and space, and programmed to re-establish tissue homeostasis [53,54,55]. However, a default in proresolving pathways can result in chronic low-grade inflammation, leading to long term excessive tissue damage and pathology [54,56]. Various mechanisms are involved in the resolution of inflammation, including the inhibition of proinflammatory markers, the increased production of anti-inflammatory cytokines and the generation of lipid metabolites called oxylipins [54,56]. We thus evaluated the effects of the fish hydrolysate on these mechanisms following systemic LPS injection. 

Dietary supplementation with fish hydrolysate reduced the LPS-induced expression of proinflammatory cytokines IL-6, IL-1β and TNF-α. We previously showed that this fish hydrolysate reduced the LPS-induced expression of IL-6 and IL-1β in microglial-like cells [44]. This beneficial effect of the fish hydrolysate can be linked, in part, to the increased protein expression of IκB that inhibits NF-κB responsible for the production of proinflammatory cytokines [57]. This decrease in the expression of proinflammatory cytokines 2 h following LPS injection was also accompanied by the reduction in CCL2 expression, which is a chemokine highly expressed by microglia to attract monocytes from the periphery to the brain during neuroinflammation [58]. Thus, our results suggest that the anti-inflammatory properties of the fish hydrolysate were sufficient to limit the recruitment of peripheral monocytes 2 h post-LPS. The immune response is orchestrated by microglial cells, which shift their functional phenotype once stimulated by inflammatory factors [59,60]. Then we evaluated the effects of the fish hydrolysate supplementation on the expression of microglial phenotype markers. Although the fish hydrolysate supplementation had no effect on the expression of the M2 phenotype markers, our results showed a higher expression of the M1 microglial activation markers CD86, CD68 and CD11b 2 h post-LPS compared to control and DHA groups. Fish hydrolysate reduced gene expression of COX-2, another marker of the M1 phenotype, also responsible for the biosynthesis of the first lipid mediators synthesized following LPS injection, the proinflammatory oxylipins (prostaglandins and leukotrienes). COX-2 expression is rapidly increased under inflammatory conditions and is regulated by NF-κB [61,62,63,64,65]. Here, we can hypothesize that 2 h-post LPS, proinflammatory oxylipins have already been produced in the fish hydrolysate supplemented mice, and that there has been a switch in lipid mediator class towards the synthesis of anti-inflammatory mediators and proresolving mediators (lipoxins and SPMs), suggesting that the resolution of inflammation has begun. 

Among the fish hydrolysate compounds, low molecular weight peptides and DHA can explain the effects described above since both have been reported to have beneficial effects on inflammation. Indeed, protein hydrolysates show strong immunomodulatory effects in animals. Oral administration of a bioactive milk tripeptide inhibits the expression of inflammatory factors such as TNF-α, CCL2 or iNOS in the hippocampus of the Alzheimer’s disease mice model [39]. In another study, milk-derived tripeptides decrease proinflammatory cytokines IL-1β, IL-6 as well as SOCS3 in the abdominal aorta of ApoE-deficient mice, thus reducing atherosclerosis development [37]. Moreover, egg-derived tripeptides significantly decrease the plasmatic expression of IL-6 and CCL2 in a model of hypertensive rats [36]. At the cellular level, peptides from salmon exerted anti-inflammatory activities by reducing TNF-α, IL-1β, IL-6 expressions and decreasing NO and PGE2 production in murine macrophages [41]. In human monocytes, an octapeptide from lupine reduces the LPS-induced expression of IL-6, TNF-α, IL-1β as well as CCL2 and increases the expression of the anti-inflammatory cytokine IL-10 [42]. 

The beneficial effect of DHA, the second compound of interest in the fish hydrolysate, has been demonstrated in vivo in numerous studies. DHA and/or EPA limit or prevent the LPS-induced increase in proinflammatory cytokines in the CNS [20,66,67,68,69]. Furthermore, mice fed with a diet enriched in α-linolenic acid, the precursor of DHA, have higher brain DHA levels and produce less cortical proinflammatory cytokines and CCL2 24h post traumatic brain injury [70]. These beneficial effects of DHA on the CNS or the brain could be direct or indirect. Indeed, DHA can act directly by downregulating the activation of NF-κB, p38 MAPK as well as JNK and consequently decreasing the production of proinflammatory cytokines and chemokines by microglia in vitro [67,71,72,73,74,75,76]. Moreover, DHA can reduce microglial activation and shift microglial polarization toward the M2 phenotype in vivo and in vitro under inflammatory conditions and in models of neurological diseases [66,77,78,79,80,81,82,83]. DHA can also act indirectly via the synthesis of SPMs, which reduce the intensity of inflammation [54]. SPMs include protectins, D-series resolvins, and maresins [54,84,85]. They display anti-inflammatory and proresolving properties as well as neuroprotective functions [19,68,86,87]. Dietary interventions with n-3 PUFAs, including DHA, have been shown to increase EPA- and DHA-derived oxylipins and decrease oxylipins derived from AA [20,23,24]. Conversely, Taha et al. [28] have shown that dietary n-6 PUFA supplementation increases AA-derived oxylipins and decreases EPA-derived oxylipins. Furthermore, Rey et al. [20] showed that a dietary supplementation with n-3 long chain PUFAs induces an anti-inflammatory oxylipin profile in mice 24h post-LPS. This oxylipin generation following LPS injection can explain, in part, the differential effect between fish hydrolysate and DHA supplementations. Indeed, as compared to DHA supplementation, the fish hydrolysate supplementation induced a higher production of oxylipins derived from DHA, Mar1, its precursor 14-HDoHE and 17-HDoHE, which are derived from the LOX pathway [17]. Mar1 was first identified in macrophages and is involved in the resolution of inflammation [88,89]. Mar1 has been shown to decrease proinflammatory signaling cascades and influence macrophages towards an M2 repair phenotype after cerebral ischemia or spinal cord injury [90,91,92]. The increase in 17-HDoHE and 14-HDoHE has been reported to down-regulate the inflammatory response in the CNS and in microglia in vitro [19,20,93]. In addition, 17R-HDHA and 17S-HDHA reduce the production of proinflammatory cytokines in the spinal cord and hippocampus [68,94]. Furthermore, the fish hydrolysate supplementation increased the production of LxA4, which is a lipid-mediator class switching between the acute inflammation phase and the resolution phase. It possesses anti-inflammatory properties through the ALX/Fpr2 receptor, which is particularly expressed in microglia [22,94,95,96,97]. LxA4 inhibits neutrophil infiltration, decreases proinflammatory cytokine expression via the inhibition of NF-κB, phosphorylation of p38 MAPK and JNK [98,99,100]. Furthermore, the fish hydrolysate induced an increased production of 12-HETE and 15-HETE. Despite the fact that 12- and 15-HETE are mostly described as proinflammatory, anti-inflammatory properties have also been highlighted [101]. Indeed, they can activate PPARγ, attenuate COX-2 and iNOS expression and inhibit the NF-κB pathway in ischemic rat brain [102]. Thus, our results suggest that the fish hydrolysate supplementation induced a faster transition from the acute inflammation phase to the resolution phase compared to control and DHA groups. 

Interestingly, we also demonstrated that the differential effect between the fish hydrolysate and DHA supplementation could be due to the greater attenuation of LPS-induced expression of proinflammatory cytokines of the fish hydrolysate supplementation as compared to DHA supplementation. Indeed, 2 h following LPS injection, DHA supplementation only reduced the expression of IL-6 whereas the fish hydrolysate supplementation decreased the expression of IL-6, IL-1β and TNF-α. We hypothesized that the effect on proinflammatory cytokines could be due to peptides alone or to the potentiation of DHA present in low amount in the fish hydrolysate by the low molecular weight peptides. To answer this point, we have to isolate and test separately the low molecular weight peptides. Another hypothesis would be that the fish hydrolysate modified the kinetic of the resolution of inflammation by shifting microglia polarization into a more reactive phenotype, as shown by the increase in microglial activation, which could be in accordance with a faster immune response and beneficial during acute inflammation. Altogether, our results showed that the fish hydrolysate supplementation induced an earlier immune response 2 h post-LPS as compared to DHA supplementation. Indeed, the shift of microglia into a more reactive phenotype allowed a lower inflammatory response which was associated to the decrease in proinflammatory markers and to the production of anti-inflammatory and proresolutive oxylipins. These changes could explain the different hippocampal oxylipin profile compared to control and DHA groups, and thus the modulation of the immune response observed.

## 5. Conclusions

These results are particularly of interest since the fish hydrolysate allowed a faster immune response compared to a supplementation with 100 times more DHA, described as the efficient dose in the literature. Furthermore, this fish hydrolysate was obtained through the valorization of marine byproducts in a context of sustainable development. Thus, it remains an innovative and good candidate for the prevention of inflammation and notably in the case of neurodegenerative pathologies, which are characterized by a chronic low-grade inflammation. Nevertheless, in this work we did not evaluate the impact of hydrolysate supplementation on LPS-injected mice outcome as fever, food intake and sickness behavior. Further investigations are still needed to decipher the individual role of low molecular weight peptides and DHA by realizing a time course of inflammatory markers expression after LPS injection to better understand the mechanisms of action at the cerebral level and to characterize the peptides contained in this hydrolysate. 

## Figures and Tables

**Table 1:** Composition of the supplementations.

	Control	Hydrolysate	DHA
Water (µL/day)	100	-	100
Peanut oil (µL/day)	50	50	-
Fish hydrolysate group (µL/day)	-	100	-
Peptides (mg/day)	-	5	-
DHA (mg/day)	-	0.143	-
DHA supplementation group (µL/day)	-	-	50
DHA (mg/day)	-	-	10

DHA: docosahexaenoic acid.

**Table 2:** Cortical fatty acid composition of mice fed orally with control solution, fish hydrolysate or DHA by daily gavage for 18 days, 2 h following LPS injection (125 µg/kg).

Fatty Acid (% of Total Fatty Acids)	Control		Hydrolysate		DHA		Statistical Effect		
	Saline	LPS	Saline	LPS	Saline	LPS	Supplementation	LPS	Supplementation × LPS
14:0	0.13 ± 0.00	0.13 ± 0.00	0.12 ± 0.00	0.13 ± 0.00	0.13 ± 0.00	0.13 ± 0.00	-	-	-
15:0	0.04 ± 0.00	0.05 ± 0.00	0.05 ± 0.00	0.05 ± 0.00	0.05 ± 0.01	0.04 ± 0.00	-	-	-
16:0	21.43 ± 0.22	21.36 ± 0.31	21.36 ± 0.15	21.49 ± 0.12	21.27 ± 0.07	21.10 ± 0.15	-	-	-
17:0	0.14 ± 0.00	0.16 ± 0.01	0.14 ± 0.00	0.14 ± 0.00	0.16 ± 0.02	0.14 ± 0.00	-	-	-
18:0	20.03 ± 0.06 ^a^	19.65 ± 0.04 ^b^	20.06 ± 0.08 ^a^	19.66 ± 0.17 ^b^	19.74 ± 0.05 ^b^	19.78 ± 0.09 ^b^	-	*p* = 0.0001	*p* = 0.0034
20:0	0.22 ± 0.01	0.21 ± 0.01	0.21 ± 0.01	0.21 ±0.01	0.22 ± 0.01	0.23 ± 0.01	-	-	-
22:0	0.15 ± 0.01	0.17 ± 0.02	0.16 ± 0.01	0.15 ± 0.01	0.15 ± 0.01	0.16 ± 0.00	-	-	-
24:0	0.17 ± 0.01	0.17 ± 0.01	0.17 ± 0.01	0.17 ± 0.00	0.17 ± 0.01	0.18 ± 0.01	-	-	-
**SFAs**	**42.31 ± 0.21**	**41.89 ± 0.32**	**42.27 ± 0.19**	**42.00 ± 0.15**	**41.89 ± 0.08**	**41.76 ± 0.20**	-	-	-
16:1n-9	0.15 ± 0.00	0.16 ± 0.00	0.16 ± 0.00	0.16 ± 0.00	0.15 ± 0.00	0.15 ± 0.00	*p* = 0.0047	-	-
16:1n-7	0.51 ± 0.01	0.53 ± 0.01	0.52 ± 0.00	0.53 ± 0.01	0.52 ± 0.01	0.52 ± 0.01	-	-	-
18:1t	0.03 ± 0.00 ^a^	0.03 ± 0.00 ^a^	0.04 ± 0.00 ^a^	0.04 ± 0.01 ^a^	0.04 ± 0.01 ^a^	0.07 ± 0.00 ^b^	*p* = 0.0004	-	*p* = 0.0114
18:1n-9	14.04 ± 0.11	14.17 ± 0.11	14.17 ± 0.11	14.12 ± 0.10	14.22 ± 0.18	14.42 ± 0.11	-	-	-
18:1n-7	3.51 ± 0.01	3.55 ± 0.04	3.53 ± 0.02	3.50 ± 0.02	3.49 ± 0.03	3.49 ± 0.01	-	-	-
20:1n-9	0.90 ± 0.03	0.94 ± 0.04	0.90 ± 0.03	0.90 ± 0.03	0.93 ± 0.06	0.96 ± 0.03	-	-	-
20:1n-7	0.24 ± 0.01	0.26 ± 0.01	0.25 ± 0.01	0.24 ± 0.01	0.26 ± 0.02	0.26 ± 0.01	-	-	-
22:1n-9	0.09 ± 0.01	0.11 ± 0.01	0.09 ± 0.00	0.10 ± 0.00	0.10 ± 0.01	0.10 ± 0.00	-	-	-
24:1n-9	0.20 ± 0.01 ^a,b,c^	0.18 ± 0.01 ^b,e^	0.22 ± 0.01 ^a,d^	0.20 ± 0.01 ^b,d,e^	0.20 ± 0.01 ^a,e^	0.23 ± 0.01 ^c,d^	-	-	*p* = 0.0473
**MUFAs**	**19.68 ± 0.15**	**19.93 ± 0.18**	**19.86 ± 0.16**	**19.79 ± 0.15**	**19.90 ± 0.28**	**20.19 ± 0.14**	-	-	-
18:2n-6	0.52 ± 0.01	0.47 ± 0.02	0.51 ± 0.01	0.52 ± 0.01	0.54 ± 0.01	0.52 ± 0.01	-	-	-
20:2n-6	0.13 ± 0.00 ^a^	0.12 ± 0.00 ^b^	0.12 ± 0.00 ^b,c^	0.13 ± 0.00 ^a,b^	0.13 ± 0.00 ^a^	0.13 ± 0.00 ^a,c^	-	*p* = 0.0432	*p* = 0.0089
20:3n-6	0.41 ± 0.01	0.39 ± 0.01	0.42 ± 0.00	0.42 ± 0.01	0.47 ± 0.01	0.46 ± 0.01	*p* < 0.0001	-	-
20:4n-6	9.98 ± 0.11	10.15 ± 0.08	9.96 ± 0.09	9.95 ± 0.06	9.34 ± 0.16	9.43 ± 0.09	*p* < 0.0001	-	-
22:4n-6	2.05 ± 0.03	2.17 ± 0.07	2.05 ± 0.02	2.14 ± 0.04	1.93 ± 0.04	2.02 ± 0.04	*p* = 0.0069	*p* = 0.0218	-
22:5n-6	0.30 ± 0.01	0.32 ± 0.02	0.29 ± 0.01	0.30 ± 0.01	0.28 ± 0.02	0.27 ± 0.00	*p* = 0.0001	-	-
**n-6**	**13.41 ± 0.13**	**13.61 ± 0.16**	**13.34 ± 0.08**	**13.45 ± 0.07**	**12.69 ± 0.19**	**12.81 ± 0.13**	*p* < 0.0001	-	-
20:5n-3	0.06 ± 0.01	0.06 ± 0.00	0.06 ± 0.00	0.06 ± 0.00	0.08 ± 0.00	0.08 ± 0.00	*p* < 0.0001	-	-
22:5n-3	0.13 ± 0.00	0.14 ± 0.00	0.14 ± 0.00	0.14 ± 0.00	0.17 ± 0.00	0.17 ± 0.00	*p* < 0.0001	-	-
22:6n-3	15.85 ± 0.29	15.67 ± 0.25	15.69 ± 0.24	16.09 ± 0.24	16.74 ± 0.28	16.19 ± 0.18	*p* = 0.0175	-	-
**n-3**	**16.05 ± 0.29**	**15.87 ± 0.24**	**15.89 ± 0.24**	**16.29 ± 0.24**	**16.99 ± 0.28**	**16.43 ± 0.18**	*p* = 0.01	-	-
20:3n-9	0.13 ± 0.02 ^a,b^	0.09 ± 0.01 ^c,d^	0.17 ± 0.03 ^a^	0.08 ± 0.00 ^c,d^	0.10 ± 0.00 ^b,c^	0.12 ± 0.01 ^b,d^	-	*p* = 0.0066	*p* = 0.0122
**PUFAs**	**29.59 ± 0.31**	**29.56 ± 0.28**	**29.40 ± 0.26**	**29.81 ± 0.20**	**29.78 ± 0.38**	**29.36 ± 0.13**	-	-	-
dma16:0	2.23 ± 0.03	2.36 ± 0.03	2.27 ± 0.03	2.27 ± 0.05	2.24 ± 0.05	2.28 ± 0.02	-	-	-
dma18:0	3.99 ± 0.03	3.99 ± 0.03	3.98 ± 0.03	3.92 ± 0.03	3.97 ± 0.05	4.04 ± 0.03	*p* = 0.0448	-	-
dma18:1n-9	1.19 ± 0.02	1.22 ± 0.03	1.20 ± 0.03	1.19 ± 0.02	1.19 ± 0.03	1.24 ± 0.02	-	-	-
dma18:1n-7	1.02 ± 0.02	1.05 ± 0.03	1.03 ± 0.02	1.03 ± 0.03	1.04 ± 0.03	1.08 ± 0.02	-	-	-
**DMA**	**8.43 ± 0.08**	**8.62 ± 0.08**	**8.48 ± 0.06**	**8.41 ± 0.13**	**8.43 ± 0.15**	**8.64 ± 0.09**	-	-	-
**n-6/n-3**	**0.84 ± 0.02**	**0.86 ± 0.02**	**0.84 ± 0.01**	**0.83 ± 0.01**	**0.75 ± 0.01**	**0.78 ± 0.01**	*p* < 0.0001	-	-

Values with superscripts (a, b, c, d, e) differ significantly, n = 4–6/group. Data are presented as mean ± SEM and are expressed as percentage of total fatty acids. DHA: docosahexaenoic acid; DMA: dimethylacetals; LPS: lipopolysaccharide; MUFAs: monounsaturated fatty acids; PUFAs: polyunsaturated fatty acids; SFAs: saturated fatty acids.

**Table 3:** Hippocampal oxylipin concentration of mice fed orally with control solution, fish hydrolysate or DHA by daily gavage for 18 days, 2 h following LPS injection (125 µg/kg).

Oxylipin (pg /mg protein)	Control		Hydrolysate		DHA		Statistical Effect		
	Saline	LPS	Saline	LPS	Saline	LPS	Supplementation	LPS	Supplementation × LPS
**AA-oxylipins**									
**Thromboxanes, prostaglandins**									
TxB2	1584.36 ± 136.93	1739.60 ± 138.94	1729.72 ± 220.19	1495.16 ± 30.78	1781.36 ± 166.54	1747.47 ± 205.07	-	-	-
6kPGF1α	151.68 ± 68.79 ^a^	505.96 ± 66.67 ^b^	246.65 ± 83.26 ^a^	78.59 ± 49.27 ^a^	210.93 ± 69.00 ^a^	226.75 ± 92.39 ^a^	-	-	*p* = 0.0086
PGF2α	4429.79 ± 380.97	5550.45 ± 330.52	4582.04 ± 486.14	5708.87 ± 577.25	5656.33 ± 461.46	5155.75 ± 727.26	-	-	-
PGE2	1428.02 ± 141.17 ^a^	2366.55 ± 121.53 ^b^	1410.87 ± 117.93 ^a^	2356.64 ± 224.38 ^b^	1883.66 ± 204.02 ^a,b^	1881.02 ± 257.90 ^a,b^	-	*p* = 0.0003	*p* = 0.0211
PGD2	1741.08 ± 319.14 ^a^	3208.60 ± 445.99 ^b^	1639.05 ± 216.33 ^a^	3188.42 ± 282.95 ^b,c^	2301.83 ± 244.21 ^a,b^	2178.11 ± 411.06 ^a,c^	-	*p* = 0.002	*p* = 0.0314
PGA1	0.00 ± 0.00 ^a^	28.65 ± 7.78 ^b^	0.00 ± 0.00 ^a^	34.75 ± 11.80 ^b^	0.00 ± 0.00 ^a^	0.00 ± 0.00 ^a^	*p* = 0.0014	*p* < 0.0001	*p* = 0.0014
15dPGJ2	31.15 ± 19.37	71.23 ± 8.44	13.59 ± 13.59	89.69 ± 19.08	45.69 ± 23.73	53.66 ± 24.59	-	*p* = 0.0145	-
8isoPGA2	464.81 ± 24.87	651.95 ± 76.86	500.41 ± 109.57	864.62 ± 133.33	682.20 ± 116.56	750.64 ± 92.68	-	*p* = 0.0184	-
**Lipoxins**									
LxA4	265.31 ± 31.85 ^a^	764.10 ± 117.00 ^c^	352.61 ± 37.66 ^a,b^	1126.31 ± 115.00 ^d^	631.05 ± 121.16 ^b,c^	614.25 ± 151.20 ^b,c^	-	*p* < 0.0001	*p* = 0.0041
LxB4	119.60 ± 79.81	413.04 ± 100.91	488.09 ± 79.65	560.64 ± 67.12	342.60 ± 115.89	309.99 ± 112.83	*p* = 0.0483	-	-
**Epoxy fatty acids**									
14,15-EET	258.15 ± 55.32	218.68 ± 27.43	221.62 ± 44.03	339.82 ± 95.94	317.46 ± 68.49	321.12 ± 36.59	-	-	-
8,9-EET	665.74 ± 86.14	717.35 ± 62.99	745.55 ± 88.82	1132.12 ± 251.53	984.20 ± 127.64	904.92 ± 119.39	-	-	-
5,6-EET	510.40 ± 59.39	552.36 ± 22.88	607.26 ± 61.65	776.15 ± 152.98	730.88 ± 94.28	687.14 ± 90.15	-	-	-
**Hydroxy fatty acids**									
15-HETE	5381.63 ± 491.85 ^a^	10236.55 ± 1133.60 ^b,c^	4986.08 ± 394. 85 ^a^	12370.92 ± 946.68 ^c^	8412.97 ± 1033.41 ^b^	8271.61 ± 1124.26 ^b^	-	*p* < 0.0001	*p* = 0.0014
8-HETE	616.39 ± 59.62 ^a^	861.88 ± 71.18 ^b^	536.21 ± 32.63 ^a^	1094.93 ± 100.61 ^c^	825.45 ± 81.77 ^b^	823.81 ± 84.78 ^b^	-	*p* = 0.0001	*p* = 0.0033
12-HETE	1496.97 ± 53.35 ^a,b^	2662.28 ± 394.98 ^c,d^	1188.01 ± 51.49 ^a^	3479.62 ± 252.58 ^d^	2076.89 ± 258.48 ^b,c^	2145.92 ± 439.72 ^b,c^	-	*p* < 0.0001	*p* = 0.0044
5-HETE	5518.82 ± 671.54	6747.00 ± 403.13	5484.87 ± 486.33	8002.11 ± 382.00	7715.60 ± 1005.41	8939.82 ± 1212.00	*p* = 0.0318	*p* = 0.0262	-
5-oxoETE	5524.57 ± 639.67 ^a^	6300.15 ± 474.39 ^a,c^	5286.41 ± 199.95 ^a^	9143.13 ± 785.06 ^b^	8623.43 ± 291.86 ^b^	7449.52 ± 981.74 ^b,c^	*p* = 0.0072	*p* = 0.0342	*p* = 0.0022
**LA-oxylipins**									
**Hydroxy fatty acids**									
13-HODE	1294.54 ± 41.33 ^a^	2091.36 ± 184.04 ^a^	1429.20 ± 192.49 ^a^	3655.32 ± 871.76 ^b^	2266.32 ± 315.64 ^a^	2212.73 ± 339.80 ^a^	-	*p* = 0.0026	*p* = 0.0134
9-HODE	497.38 ± 19.99 ^a^	979.03 ± 109.81 ^b,c^	577.19 ± 76.69 ^a,b^	1628.82 ± 355.30 ^d^	967.76 ± 132.13 ^b,c^	950.78 ± 147.61 ^a,c^	-	*p* = 0.0005	*p* = 0.0066
**DHA-oxylipins**									
**Hydroxy fatty acids**									
17-HDoHE	1208.83 ± 91.11 ^a,b^	2030.94 ± 216.72 ^c,d^	1002.70 ± 83.64 ^b^	2428.07 ± 154.48 ^c^	1717.12 ± 318.00 ^a,d^	1731.76 ± 194.06 ^a,d^	-	*p* = 0.0001	*p* = 0.0076
14-HDoHE	819.92 ± 71.54 ^a,b^	1171.61 ± 168.81 ^b,c^	639.76 ± 45.18 ^a^	1683.52 ± 143.81 ^d^	1266.36 ± 167.27 ^c,d^	1290.20 ± 175.01 ^c,d^	-	*p* = 0.0004	*p* = 0.0056
7-MaR1	0.00 ± 0.00 ^a,b^	42.73 ± 11.82 ^b^	0.00 ± 0.00 ^a^	88.89 ± 50.81 ^c^	0.00 ± 0.00^a^	0.00 ± 0.00^a^	*p* = 0.0289	*p* = 0.0019	*p* = 0.0289

Values with superscripts (a, b, c, d) differ significantly, n = 4–6/group. Data are presented as mean ± SEM and are expressed in pg/mg protein. 5-oxoETE: 5-oxo-eicosatetraenoic; 7-MaR1: 7(S)-maresin; AA: arachidonic acid; DHA: docosahexaenoic acid; EET: epoxy-eicosatrienoic acid; HDoHE: hydroxy-docosahexaenoic acid; HETE: hydroxy-eicosatetraenoic acid; HODE: hydroxy-octadecadienoic acid; LA: linoleic acid; LPS: lipopolysaccharide; Lx: lipoxin; PG: prostaglandin; TxB2: thromboxane B2.

## Data Availability

The data presented in this study are available on request from the corresponding author.

## Abbreviations

5-oxoETE	5-oxo-eicosatetraenoic acid
AA	arachidonic acid
ALA	α-linolenic acid
Arg1	arginase 1
BDNF	brain-derived neurotrophic factor
CD	cluster of differentiation
CNS	central nervous system
COX	cyclooxygenase
DHA	docosahexaenoic acid
EET	epoxy eicosatrienoic acid
EPA	eicosapentaenoic acid
HDoHE	hydroxy-docosahexaenoic acid
HETE	hydroxy-eicosatetraenoic acid
HODE	hydroxy-octadecadienoic acid
IL	interleukin
iNOS	inducible nitric oxide synthase
JNK	c-Jun N-terminal kinase
LA	linoleic acid
LC-PUFAs	long chain polyunsaturated fatty acids
LOX	lipoxygenase
LPS	lipopolysaccharide
Lx	lipoxin
MAPK	mitogen-activated protein kinase
MaR	maresin
MCP-1/CCL2	monocyte chemoattractant protein-1
NF-κB	nuclear factor kappa B
NGF	nerve growth factor
NO	nitric oxide
PG	prostaglandin
PUFAs	polyunsaturated fatty acids
SPMs	specialized proresolving mediators
SOCS	Suppressor of cytokine signalling
TGF	transforming growth factor
TLR	toll-like receptor
TNF	tumor necrosis factor
Trk	tyrosine receptor kinase
Tx	thromboxane
